# Temperature-Dependent Dynamical Evolution in Coum/SBE-β-CD Inclusion Complexes Revealed by Two-Dimensional FTIR Correlation Spectroscopy (2D-COS)

**DOI:** 10.3390/molecules26123749

**Published:** 2021-06-19

**Authors:** Giuseppe Paladini, Francesco Caridi, Vincenza Crupi, Federica De Gaetano, Domenico Majolino, Silvana Tommasini, Cinzia Anna Ventura, Valentina Venuti, Rosanna Stancanelli

**Affiliations:** 1 Dipartimento di Scienze Matematiche e Informatiche, Scienze Fisiche e Scienze della Terra, Università degli Studi di Messina, Viale Ferdinando Stagno D’Alcontres 31, 98166 Messina, Italy; gpaladini@unime.it (G.P.); fcaridi@unime.it (F.C.); vvenuti@unime.it (V.V.); 2 Dipartimento di Scienze Chimiche, Biologiche, Farmaceutiche e Ambientali, Università degli Studi di Messina, Viale Ferdinando Stagno D’Alcontres 31, 98166 Messina, Italy; vcrupi@unime.it (V.C.); fedegaetano@unime.it (F.D.G.); stommasini@unime.it (S.T.); rstancanelli@unime.it (R.S.)

**Keywords:** inclusion complex, temperature effect, FTIR-ATR, 2D correlation spectroscopy, time sequence, molecular motions

## Abstract

A combination of Fourier transform infrared spectroscopy in attenuated total reflectance geometry (FTIR-ATR) and 2D correlation analysis (2D-COS) was applied here for the first time in order to investigate the temperature-dependent dynamical evolution occurring in a particular type of inclusion complex, based on sulfobutylether-β-cyclodextrin (SBE-β-CD) as hosting agent and Coumestrol (7,12-dihydorxcoumestane, Coum), a poorly-soluble active compound known for its anti-viral and anti-oxidant activity. For this purpose, synchronous and asynchronous 2D spectra were calculated in three different wavenumber regions (960–1320 cm^−1^, 1580–1760 cm^−1^ and 2780–3750 cm^−1^) and over a temperature range between 250 K and 340 K. The resolution enhancement provided by the 2D-COS offers the possibility to extract the sequential order of events tracked by specific functional groups of the system, and allows, at the same time, the overcoming of some of the limits associated with conventional 1D FTIR-ATR analysis. Acquired information could be used, in principle, for the definition of an optimized procedure capable to provide high-performance *T*-sensitive drug carrier systems for different applications.

## 1. Introduction

Over the last years, many efforts have been devoted to the design of high-performance drug delivery systems capable to transport suitable dosage of the drug with high spatial and temporal accuracy. As is well-known, drugs administered through conventional routes may lead to a series of unwanted effects mainly induced by their poor water solubility in lipophilic solvents and insufficient bioavailability [1,2,3,4,5,6,7], strongly limiting their pharmacological efficacy. In this context, supramolecular compounds based on natural and modified cyclodextrins (CDs and MCDs, respectively) represent an attractive strategy for the engineering of bio-friendly host-guest inclusion complexes capable to improve the chemical/physical properties of pharmaceutical agents both in solution or in solid state [8,9,10,11,12]. Natural CDs are macrocyclic oligomers consisting of repetitive α-D-glucose units linked together through α-1,4-glycosidic linkages in such a way to arrange a toroidal-shaped structure. According to the number of α-D-glucose units, α- (6 units), β- (7 units) and γ-CDs (8 units) can be distinguished, all characterized by a polar exterior surface (where most of the free hydroxyl groups fall) and a non-polar (hydrophobic) inner cavity. Hydrophobicity of CDs cavities certainly arises from the presence of both oxygen and hydrogen atoms involved in inter-unit glycosidic bonds, allowing “small” non-soluble drug molecules (guest) to accommodate within the CD cavity (host) through the establishment of non-covalent host-guest interactions (i.e., Van der Waals forces, electrostatic interactions and hydrogen bonds (HBs)).

Drug carriers holding therapeutical agents exhibit a series of advantageous physico-chemical/mechanical properties if compared to the drug in the pure form, including: (i) improved dissolution rates, bioavailability and aqueous solubility [13]; (ii) reduced tendency to develop drug-drug interactions [14]; and (iii) enhanced spatial/temporal release control of biologically active compounds [15,16]. In this sense, additional benefits can be achieved by the use of MCDs, derivatives of natural CDs, whose application for pharmaceutical/medical therapeutic treatments has been extensively studied and formally approved by the U.S. Food and Drug Administration (FDA). More specifically, regioselective alkylation and acylation of CDs’ hydroxyl species with, for example, hydroxypropyl (HP) [17,18,19,20], dimethyl (DM) [21,22] and sulfobutylether (SBE) groups [23], allow the development of MCDs characterized by higher performances with respect to the corresponding parental forms. For example, SBE-β-CDs were found to be more soluble when immersed in lipophilic solvents with respect to their parental formulations (β-CDs), passing from 1.85 g/100 mL at 25 °C for β-CDs up to 70 g/100 mL at 25 °C for SBE-β-CDs [24]. The polyanionic nature of SBE-β-CDs allows the synthetization of stable complexes with a large variety of insoluble drugs, positively affecting their pharmaco-kinetics properties and dynamical response.

**Figure 1:**
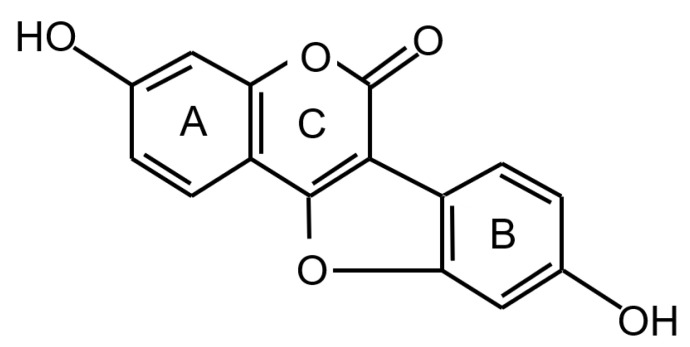
Chemical structure of coumestrol.

Coumestrol (7,12-dihydorxcoumestane, Coum, Figure 1) is a phytoestrogen typically contained in sunflower seeds, vanilla soy milk, alfalfa sprouts and leguminous plants, whose therapeutic effects have been largely investigated in the literature [25,26,27,28,29,30,31].

They demonstrate beneficial responses against heart-, coronary- and artery-related diseases, and extensive antioxidant, antimicrobial and antiestrogenic capability for different therapeutic applications [32]. This active compound also prevents the occurrence of 3α-HSD and aromatase activities in humans [33,34] as well as of the 20α-HSDH enzyme [35], respectively, involved in the progression of affective disorders and progesterone homeostasis. In addition, Coum has been found to exhibit neuroprotective effects upon cerebral ischemia (in rats) [36] and constitutes a promising solution as a pharmaceutical agent for cancer chemo-prevention [37]. It is worth noting, however, that its low aqueous solubility and reduced dissolution rate make its administration for therapeutical treatments not always feasible. To overcome both these limitations, complexation of the pure drug with natural or modified CDs became necessary.

As well-established [38,39,40,41], the complexation mechanism of inclusion complexes can be properly assessed through Fourier transform infrared spectroscopy in attenuated total reflectance geometry (FTIR-ATR), starting from the analysis of the host-guest interactions occurring at molecular level. Previous 1D FTIR-ATR spectroscopy studies [42,43] conducted on Coum/β-CD, Coum/HP-β-CD and Coum/SBE-β-CD inclusion complexes (1:1 M ratio) revealed how the establishment and/or changes in polar bonds play the main role in the complexation process. As a first prosecution of the aforementioned studies, in this work, we employed 2D correlation spectroscopy (2D-COS) FTIR-ATR technique in order to better highlight the influence of temperature on the dynamical properties exhibited by Coum/SBE-β-CD inclusion compounds. The enhanced spectral resolution achieved by 2D-COS allowed us to evaluate, upon variations in the external variable (*T*), the existence of correlations between perturbation-activated spectral responses. In addition, starting from the analysis of the 2D spectral intensity variations in the so-called synchronous (2DSMs) and asynchronous (2DAMs) maps, the sequential order of changes affecting different functional groups involved in the complexation phenomena is also shown. Both aspects are, in turn, strongly affected by the existence of different relative rate of spectral variations upon external stimuli (*T*, in our case), providing information about the progression followed by the system during a transient process, as well as the occurrence of intermolecular interactions between material’s components.

It is worth noting that the partial overlap of several vibrational bands ascribed to both Coum and SBE-β-CD molecules makes the definition of an unambiguous assignment of each spectral feature and its evolution vs. *T* difficult to accomplish by 1D FTIR-ATR spectroscopy. In this context, 2D-COS analysis overcomes this limitation, furnishing information on the time-step sequence of events affecting the molecular dynamics of SBE-β-CD-based inclusion complexes, not accessible through conventional analysis. Hence, knowledge of the aforementioned additional aspects could furnish useful insights for the development of next-generation CD-based drug carriers providing, at the same time, a detailed scenario of the Coum/SBE-β-CD complex behavior when exposed to temperature perturbations.

## 2. Results and Discussion

**Figure 2:**
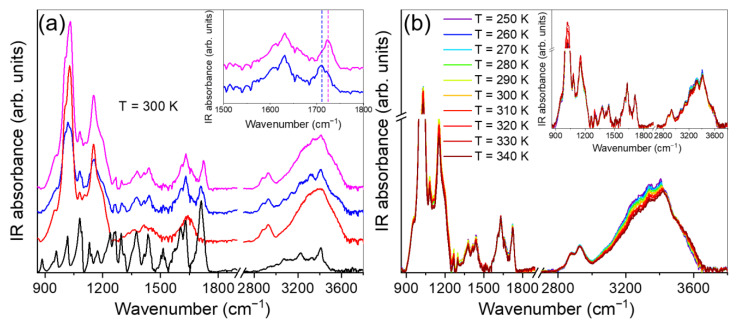
(**a**) Experimental 1D FTIR-ATR spectra, collected in the 850–3800 cm^−1^ wavenumber range at *T* = 300 K, of Coum (black line), SBE-β-CD (red line), Coum+SBE-β-CD physical mixture (blue line) and Coum/SBE-β-CD inclusion complex (purple line). In the inset, a comparison between the FTIR-ATR spectra of Coum+SBE-β-CD physical mixture (blue line) and Coum/SBE-β-CD inclusion complex (purple line) is displayed in the 1500–1800 cm^−1^ region, where the characteristic C=O stretching vibration of Coum (~1710–1725 cm^−1^) falls. (**b**) Evolution versus *T*, in the 850–3800 cm^−1^ wavenumber region, of Coum/SBE-β-CD inclusion complex and, in the inset, Coum+SBE-β-CD physical mixture 1D FTIR-ATR spectra.

As already reported [42], the complexation process occurring between Coum and SBE-β-CD was testified by dissimilarities in the experimental 1D FTIR-ATR spectra of the Coum/SBE-β-CD inclusion complex with respect to the corresponding Coum+SBE-β-CD physical mixture (a simple blending in 1:1 M ratio of the guest and host molecules), as well as individual components (see Figure 2a). The observed spectral variations were interpreted in terms of the activation of host-guest interactions involving specific and localized functional groups, particularly evident in the region between 1500–1800 cm^−1^ and 2700–3800 cm^−1^, where the characteristic C=O (1500–1800 cm^−1^), C–H and O–H (2700–3800 cm^−1^) stretching vibrations fall. Now, it is worth noting that the presence of host-guest interactions can be considered as responsible for the establishment of several correlations between couples of perturbation-induced spectral responses that are reasonably not observable in physical mixture counterparts. In this sense, differences in the 2D correlation maps between the inclusion complex and the corresponding physical mixture should be expected. Based on these considerations, we focused on the dynamical behavior exhibited by the Coum/SBE-β-CD material upon thermal stimuli, resulting from the application of an upward temperature trajectory, that follows a well-defined “time” sequence that cannot be highlighted by conventional 1D FTIR-ATR analyses.

Figure 2b depicts the evolution versus *T*, in the 250–340 K range, of the experimental 1D FTIR-ATR spectra of the Coum/SBE-β-CD inclusion complex and, in the inset of the same figure, Coum+SBE-β-CD physical mixture in the wavenumber range between 850 and 3800 cm^−1^, already discussed elsewhere [42]. The 1D FTIR-ATR spectra shown in Figure 2b were utilized to generate the 2DSMs and 2DAMs.

A preliminary assessment of all the material vibrational modes is required in order to properly encode, by 2D-COS, the time-sequence of events affecting the Coum/SBE-β-CD molecular motions.

Briefly, by recalling well-known assignments already reported in the literature [39,42,43,44,45,46], the 900–1450 cm^−1^ wavenumber region (see Figure 2b) shows contributions at ~990 cm^−1^ (shoulder), ~1020 cm^−1^, ~1080 cm^−1^ and ~1150 cm^−1^, ascribed to the =CH bending (of terminal moieties), C–O stretching, C–O–C stretching and C–H stretching vibrations of the SBE-β-CD macrocycle, respectively. Furthermore, peaks at ~1260 cm^−1^, ~1305 cm^−1^, ~1370 cm^−1^ and ~1436 cm^−1^ can be ascribed to the C–O stretching, C–O–C stretching, C–OH bending and C–C skeletal stretching vibrations of Coum molecules. In the 1500–1700 cm^−1^ wavenumber region, the most intense contributions fall at ~1601 cm^−1^, ~1629 cm^−1^ and ~1715 cm^−1^, respectively assigned to the phenyl C=C (the first two) and ketone C=O (the third one) stretching vibrations of the guest aromatic rings. The latter, as can be observed from the inset of Figure 2a, experienced an intense high-frequency shift as a consequence of the complexation, indicating the direct involvement of the aforementioned group in the inclusion complex formation [42,43]. The aforementioned region also comprises the large, almost completely convoluted, band at ~1639 cm^−1^, assigned to the δ-HOH bending of crystallization water molecules directly attached to the SBE-β-CD cyclical segments.

As far as the high-frequency region of the 1D FTIR-ATR spectra is concerned, the large band between ~2700 cm^−1^ and ~3000 cm^−1^ can be assigned to the –CH and –CH_2_ stretching vibrations of both SBE-β-CD and Coum molecules, while the large 3000–3700 cm^−1^ wavenumber band comprises all the OH stretching modes coming from primary/secondary hydroxyl groups of cyclodextrin, pure guest and differently-arranged water molecules, respectively.

**Figure 3:**
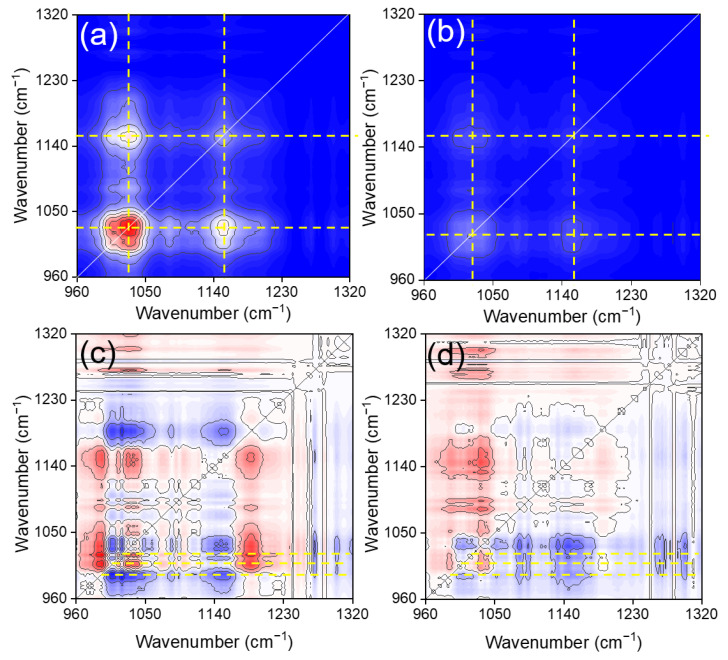
2DSM and 2DAM for the Coum/SBE-β-CD inclusion complex ((**a**,**c**), respectively), and corresponding physical mixture ((**b**,**d**), respectively), in the 960–1320 cm^−1^ wavenumber range. In the case of 2DSMs, positive cross-correlation intensities ($\Phi \left(\nu_{1},\nu_{2}\right)>0$) are marked in red, while null ones in blue ($\Phi \left(\nu_{1},\nu_{2}\right)=0$). On the other side, in 2DAMs, red, blue and white colored regions indicate positive, negative and null cross-correlation intensities, respectively. Yellow dashed lines are guide for the eyes. See text for details.

Figure 3 depicts the 2DSMs and 2DAMs for the Coum/SBE-β-CD inclusion complex and Coum+SBE-β-CD physical mixture in the 960–1320 cm^−1^ wavenumber range, over a temperature range between 250 K and 340 K (250 K used as reference).

The 2DSM of the Coum/SBE-β-CD inclusion complex (Figure 3a) highlights the presence of an intense auto-peak (AP) at ~(1025,1025) cm^−1^ followed by a low-intensity one at ~(1155,1155) cm^−1^, both related to spectral features which experience major changes upon variations of the perturbation variable (*T*, in our case). In addition, considering the area below the diagonal line for which $\nu_{1}>\nu_{2}$, a positive cross-peak (CP) at ~(1155,1025) cm^−1^ can be identified, indicating that signals at ~1155 cm^−1^ and ~1025 cm^−1^, associated to the C–H and C–O stretching vibrations of the SBE-β-CD macrocycle, change towards the same “direction”, following a uniform trend (they both decrease). The absence of correlation “squares”, except for the one formed by the previously discussed points, leads us to conclude that the remaining vibrational modes within the 960–1320 cm^−1^ wavenumber range exhibit a strong asynchronous progression as *T* increases. In the case of the 2DSM of Coum+SBE-β-CD physical mixture (Figure 3b), all the in-phase correlations observed in the 2DSM of the inclusion complex are almost vanished, indicating an almost null susceptibility of the C–H and C–O modes upon heating.

**Figure 4:**
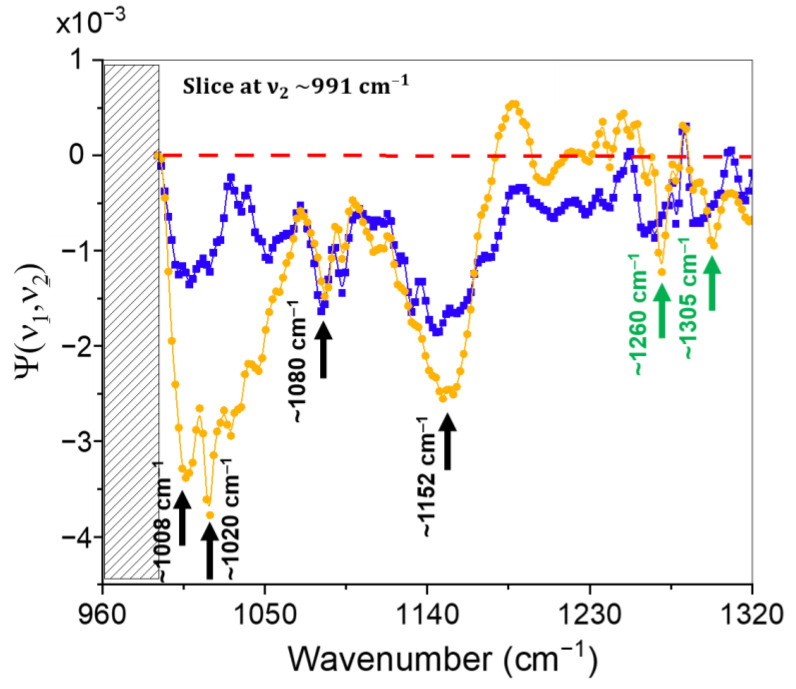
Asynchronous correlation intensity profile calculated along the horizontal slice characterized by $\nu_{2}\sim \;991{\;\mathrm{cm}}^{-1}$ for Coum/SBE-β-CD inclusion complex (orange circles) and Coum+SBE-β-CD physical mixture (blue squares). The red dashed line accounts for the $\Psi \left(\nu_{1},\nu_{2}\right)\sim 0$ condition. Thus, positive and negative correlations develop above and below this line. Considering the region of the 2DAM which satisfies the $\nu_{1}>\nu_{2}$ condition (see text for details), information originating from the left-side portion of the 2D map with respect to the diagonal line must be excluded (black hatched bar). Black and green arrows indicate $\nu_{1}$ values of interest for our discussion.

The 2DAM of the Coum/SBE-β-CD inclusion complex in the 960–1320 cm^−1^ region (Figure 3c) provides a remarkable resolution enhancement resulting from the spreading of the measured spectra over a second frequency domain. Here, both positive (red areas) and negative (blue areas) correlation regions can be recognized, suggesting a dynamical evolution of the sample functional groups characterized by different rates, following a well-defined sequential time progression. First of all, considering the region of the 2DAM that satisfies the $\nu_{1}>\nu_{2}$ condition, a horizontal slice at $\nu_{2}\;\sim 991\;{\mathrm{cm}}^{-1}$ (lowest dashed yellow line in Figure 3c), consisting of several negative asynchronous cross-correlations, can be observed. This result suggests strong asynchronicity of the aforementioned vibrational mode (~991 cm^−1^) with several other vibrational modes of both the individual components. Several cross-correlations can be also found in slices at $\nu_{2}\;\sim 1008\;{\mathrm{cm}}^{-1}$ (contribution not observable in the 1D spectra, see Figure 2b) and at $\nu_{2}\;\sim 1020{\;\mathrm{cm}}^{-1}$. To better highlight the presence of new spectroscopically-resolved contributions, as well as the sequence of events involving all the host-guest vibrational modes falling in this spectral region, Figure 4 reports the comparison of the cross-correlation intensity profile calculated along the $\nu_{2}\;\sim 991{\;\mathrm{cm}}^{-1}$ horizontal slice, as example, for both the Coum/SBE-β-CD inclusion complex and Coum+SBE-β-CD physical mixture.

First of all, as can be noted from both Figure 3c,d and Figure 4, two completely different pattern/trends for the inclusion complex and physical mixture can be recognized. The presence of sharper correlation peaks in the 2DAM of the Coum/SBE-β-CD inclusion complex (Figure 3c) with respect to those observed in the corresponding physical mixture (Figure 3d) supports the presence of constrained nano-geometries in the Coum/SBE-β-CD complexed structure [47], resulting in high-localized correlations upon thermal stimulus. The asynchronous 2D correlation slice at $\nu_{2}\;\sim 991{\;\mathrm{cm}}^{-1}$ (see Figure 4) shows strong negative-correlations with modes at ~1008 cm^−1^, ~1020 cm^−1^, ~1080 cm^−1^ and ~1152 cm^−1^ (black arrow in Figure 4). It is worth noting that the contribution falling at ~1008 cm^−1^, not clearly resolved in the 1D FTIR-ATR spectra from the band at ~1020 cm^−1^, can be associated with C–O oscillators of the macrocycle involved in a more constrained molecular environment with respect to the C–O population described by the peak at ~1020 cm^−1^.

According to the Noda’s rules [48,49], the change rate of the mode at ~991 cm^−1^ turned out to be faster, upon heating, than those observed at ~1008 cm^−1^, ~1020 cm^−1^, ~1080 cm^−1^ and ~1152 cm^−1^. This implies that the low-energy =CH bending mode of SBE-β-CD terminal moieties experiences spectral changes first, followed by the constrained and less-rigid C–O (at ~1008 cm^−1^ and ~1020 cm^−1^, respectively), C–O–C (~1080 cm^−1^), and C–H (~1152 cm^−1^) stretching vibrations of the macrocycle. The aforementioned progression reveals that temperature affects the dipole moment developed by terminal/outer units of the SBE-β-CD rims first. In fact, being the material characterized by reduced degrees of freedom as a consequence of the complexation of Coum within the SBE-β-CD nano-cavities, external functional groups of the host molecule, not strongly influenced by the establishment of host-guest interactions, “feel” temperature easier and sooner with respect to less mobile inner groups (i.e., C–O, C–O–C and C–H) [47]. Furthermore, the observation of less-intense negative-correlations with modes at ~1260 cm^−1^ and ~1305 cm^−1^ (green arrows in Figure 4), respectively associated with the C–O and C–O–C stretching vibrations of Coum, testifies that the aforementioned modes experience modifications, due to thermal motion, lastly in the proposed chronological progression. This was expected since, as already demonstrated in the literature [50], the hydrophobic nano-cavities provided by SBE-β-CD rings act as shield for embedded guests molecules against external perturbations (*T*, in our case), causing a delay of the C–O and C–O–C spectral responses of Coum.

Interestingly, in the case of the Coum+SBE-β-CD physical mixture (blue squares in Figure 4), the correlations of the aforementioned modes (C–O and C–O–C stretching of SBE-β-CD) with the peak at ~991 cm^−1^ (=CH bending mode of SBE-β-CD terminal moieties) are lost, indicating a synchronized evolution of such vibrations upon variations of the external variable (*T*). This is most likely due to the presence of not-shielded, free or self-bonded, Coum molecules, whose thermal susceptibility closely follows that of the nearby macrocycle.

**Figure 5:**
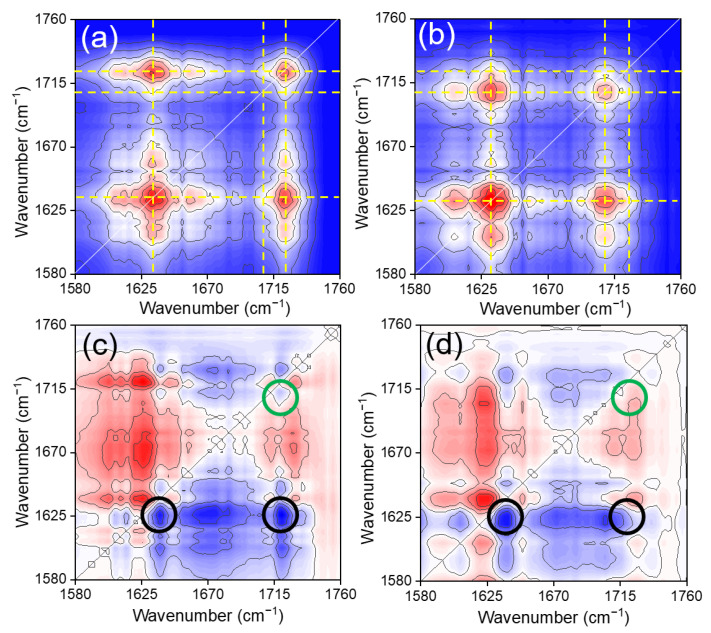
2DSM and 2DAM for the Coum/SBE-β-CD inclusion complex ((**a**,**c**), respectively), and corresponding physical mixture ((**b**,**d**), respectively), in the 1580–1760 cm^−1^ wavenumber range. In the case of 2DSMs, positive cross-correlation intensities ($\Phi \left(\nu_{1},\nu_{2}\right)>0$) are marked in red, while null ones in blue ($\Phi \left(\nu_{1},\nu_{2}\right)=0$). On the other side, in 2DAMs, red, blue and white colored regions account, respectively, for positive, negative and null cross-correlation intensities. Yellow dashed lines are guide for the eyes. The green circle in panels (**c**,**d**) identifies the CP located at ~(1724,1712) cm^−1^, while the black ones correspond to CPs located at ~(1637,1624) cm^−1^ and ~(1724,1624) cm^−1^.

In Figure 5, the 2DSMs and 2DAMs for the Coum/SBE-β-CD inclusion complex and Coum+SBE-β-CD physical mixture calculated in the 1580–1760 cm^−1^ wavenumber range, and over the 250–340 K temperature range (250 K used as reference), are displayed.

The analysis of this region, where the ketone C=O stretching band of Coum falls, appears particularly useful for the understanding the temperature-dependent behavior exhibited by the Coum/SBE-β-CD inclusion complexes upon heating, being, as stated before, the encapsulation mechanism mainly due to the establishment of intermolecular HBs between C=O functional groups of Coum and OH groups of SBE-β-CD.

A first inspection of Figure 5a reveals, in the case of the Coum/SBE-β-CD inclusion complex, the presence of two prominent APs at ~(1630,1630) cm^−1^ and ~(1722,1722) cm^−1^, indicating that the phenyl C=C (~1630 cm^−1^) and the ketone C=O (~1722 cm^−1^) stretching vibrations of the guest aromatic rings are the most affected by temperature variations. As expected, the AP at ~(1722,1722) cm^−1^ observed in Figure 5a arises consequently from the establishment of host-guest interactions involving this functional group, as it appears shifted towards lower frequencies in the 2DSM of the corresponding Coum+SBE-β-CD physical mixture (~1715 cm^−1^, see Figure 5b), in agreement with 1D FTIR-ATR results.

By looking at the 2DAMs calculated for both the inclusion complex and physical mixture (see Figure 5c,d), the appearance of both positive (red areas) and negative (blue areas) asynchronous correlation regions can be clearly observed. This occurrence furnishes an experimental evidence of an intricate progression of events involving all Coum and SBE-β-CD modes falling in this spectral range, in response to thermal motion.

In particular, the 2DAM of the Coum/SBE-β-CD inclusion complex (Figure 5c) holds three major negative CPs respectively located at ~(1724,1712) cm^−1^ (green circle in Figure 5c), ~(1637,1624) cm^−1^ and ~(1724,1624) cm^−1^ (black circles in Figure 5c). Let us initially focus the attention on the first negative asynchronous peak, ~(1724,1712) cm^−1^, which, interestingly, developed within a broad 2D spectral area that is essentially all positive. First of all, the presence of this negative asynchronous intensity testifies that the peak associated to C=O stretching vibrations of Coum (~1715 cm^−1^) is actually composed of two different contributions strongly overlapped with each other. This supports the existence of two populations of C=O oscillators involved in different molecular environments, and characterized by different, but correlated, dynamical responses upon heating. It is worth remarking that even if the presence of different classes of oscillators was already hypothesized in refs [42,43], this occurrence is evidenced here without making use of usually applied deconvolution and best-fit procedures that maintain, unfortunately, some arbitrariness. In this sense, the proposed method represents a valid way to overcome such limitations, and this result is impossible to be achieved through conventional spectroscopy. According to the literature [39,42,43], we can respectively assign the CP coordinates to the carbolyn stretching of H-bonded conjugated ketones groups (~1724 cm^−1^) of Coum molecules included inside the cyclodextrin cavity (complexed, (C=O)_complexed_), and to the carbonyl stretching of dimeric-connected Coum ones (uncomplexed, (C=O)_uncomplexed_), not involved in the host-guest bindings (~1712 cm^−1^). According to the Noda’s rules, and considering the always positive sign of the corresponding 2DSM in the same wavenumber region (see Figure 5a), we can state that the (C=O)_complexed_ (~1724 cm^−1^) groups change their conformation after the (C=O)_uncomplexed_ ones (~1712 cm^−1^) upon variations of the external variable. The proposed sequence can be interpreted in a twofold fashion.

First, carbonyl groups of guest molecules outside the cyclodextrin cavity possess a higher number of degrees of freedom with respect to that exhibited by carbonyl groups in complexed guest molecules. This means that a different mobility of C=O groups, according to whatever these groups are inside/outside the SBE-β-CD molecular cage, should be expected. In the light of these considerations, temperature was found to first affect the high-flexible/mobile carbonyl groups of uncomplexed species ((C=O)_uncomplexed_), which experience conformational changes already at the lowest temperatures of the explored *T*-range, due to a less-constrained surrounding. On the other side, the (C=O)_complexed_ groups, being associated with the guest molecules entrapped inside the cyclodextrin nano-cavities, showed a delayed spectral response reasonably ascribed to a diminishing of the guest moieties re-organization tendency, due to a more constrained environment they suffer, following the establishment of weak associations between Coum and SBE-β-CD.

In addition, the “shielding” effect provided by the investigated macrocycle should be also taken into account to explain the observed unsynchronized scenario pointed out by the ~(1724,1712) cm^−1^ CP [50,51]. In this sense, it is reasonable to suppose that, acting as a shield against temperature variations, the SBE-β-CD molecular pocket slows down the impact of an upward temperature trajectory, embracing the guest molecules against surrounding stimuli, in agreement with what has been observed from the analysis of the 960–1320 cm^−1^ wavenumber range.

Interestingly, the proposed interpretation seems to be reinforced by the fact that, by looking at the 2DAM calculated for the Coum+SBE-β-CD physical mixture, no CP at ~(1724,1712) cm^−1^ (green circle in Figure 5d) was observed. This occurrence testifies the absence of any chronological succession between the aforementioned species, indicating that, when complexation events are not expected, all C=O groups of Coum evolve in-phase with each other.

As far as the ~(1637,1624) cm^−1^ and ~(1724,1624) cm^−1^ CPs (black circles in Figure 5c) are concerned, the following sequence can be recognized: contribution at ~1624 cm^−1^, assigned to the phenyl C=C stretching vibrations of guest molecules, dynamically change upon increase in *T*, prior both the δ-HOH bending vibrations of crystallization water molecules directly attached to SBE-β-CD toroid (~1637 cm^−1^), and H-bonded carbonyl stretching of ketones groups of Coum (i.e., (C=O)_complexed_). In apparent contradiction with what has been stated in the case of the 2D-COS analysis of the ~(1724,1712) cm^−1^ CP, the phenyl ring C=C groups, which are believed to participate to the complexation phenomenon as well [39,42,43,44,45,46], are the first groups to experience dynamical changes in this chronological progression. Such discrepancy finds an explanation if we consider the most probable inclusion geometry exhibited by the investigated Coum/SBE-β-CD system. According to numerical simulations [52], the complexation pathway which led to the formation of an energy-favorable configuration for 1:1 inclusion consists in the insertion of the B-ring (see Figure 1) of Coum into the lipophilic cage of cyclodextrin through the wider rim of the macrocycle, in agreement with what also revealed for similar systems [53].

Thus, most of the C=C groups of Coum, belonging to the remaining rings, even if the complexation takes place, remains outside the cyclodextrin cavity, and, being not hindered by the entrapment in SBE-β-CD, they will consequently exhibit high susceptibility with respect to temperature stresses. Notably, since this occurrence is statistically relevant for C=C groups not properly entrapped, the detected asynchronicity turned out be relevant also for the Coum+SBE-β-CD physical mixture, as can be seen from the presence of the negative ~(1637,1624) cm^−1^ CP even in the 2DAM reported in Figure 5d (black circle on the left).

Afterwards, the amount of energy adsorbed by the system, as a consequence of a further increase in temperature, affects the δ-HOH bending vibrations of cyclodextrin water molecules (~1637 cm^−1^, not clearly resolved in 1D FTIR-ATR spectra, see inset of Figure 2a), and, finally, the last functional groups to experience fluctuations are the (C=O)_complexed_ ones (~1724 cm^−1^), confirming, once again, the protection that the macrocycle exerts on Coum against unwanted, destructuring effect of temperature. As expected, this last cross-correlation, ~(1724,1624) cm^−1^, is lost in the 2DAM of the Coum+SBE-β-CD physical mixture (see the black circle on right in Figure 5d), as the onset of a (C=O)_complexed_ population is a direct consequence of the establishment of host-guest interactions.

A 2D-COS description of the high frequency region (2780–3750 cm^−1^) was finally accomplished with the aim to determine the step-by-step evolution of the HB scheme upon heating, as well as the sequential order of events affecting all the high-energy modes. A destructuring effect of temperature on the HB pattern of Coum/SBE-β-CD complex, leading to a lowering of the overall HBs cooperativity degree, was already evidenced by the analysis of 1D FTIR-ATR spectra [42,43]. Here, however, a first attempt aimed at providing a reasonable time-progression of the dynamical behavior associated to the HB scheme upon heating is proposed.

**Figure 6:**
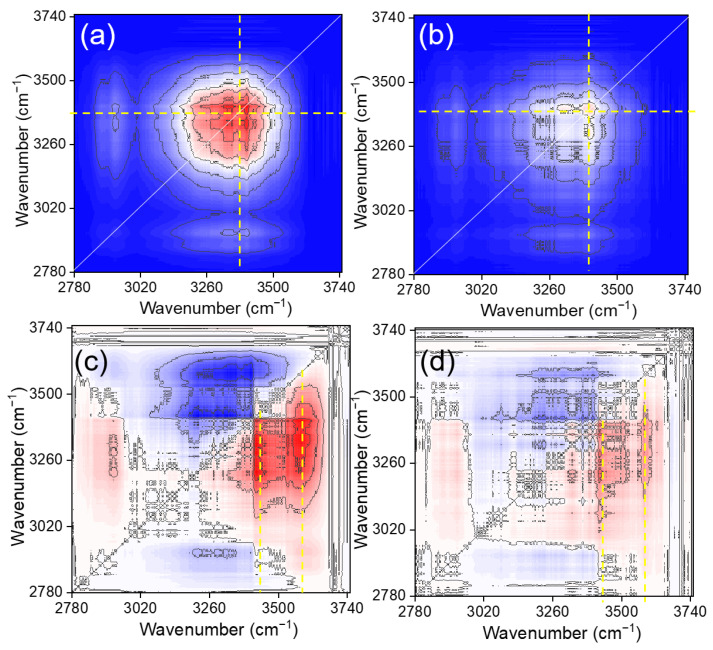
2DSM and 2DAM for the Coum/SBE-β-CD inclusion complex ((**a**,**c**), respectively), and corresponding physical mixture ((**b**,**d**), respectively), in the 2780–3750 cm^−1^ wavenumber range. In the case of 2DSMs, positive cross-correlation intensities ($\Phi \left(\nu_{1},\nu_{2}\right)>0$) are marked in red, while null ones in blue ($\Phi \left(\nu_{1},\nu_{2}\right)=0$). On the other side, in 2DAMs, red, blue and white colored regions account, respectively, for positive, negative and null cross-correlation intensities. Yellow dashed lines are guide for the eyes. See text for details.

In Figure 6, we report the 2DSMs and 2DAMs for the Coum/SBE-β-CD inclusion complex and Coum+SBE-β-CD physical mixture calculated in the 2780–3750 cm^−1^ wavenumber range, and over the 250–340 K temperature range (250 K used as reference).

A first inspection of Figure 6a,b reveals, for both the Coum/SBE-β-CD inclusion complex and Coum+SBE-β-CD physical mixture, the presence of a broad AP centered at ~(3370,3370) cm^−1^. Briefly, since the static variations of the O–H stretching band upon heating are well-documented [20,45,54,55], the difference in the observed synchronous intensity can be ascribed to the establishment, upon complexation, of inter-mode correlations involving hydroxyl groups of both individual components. It is worth noting, however, that extended high-intensity APs, like those observed in 2DSMs, develop only in the case of the absence of simultaneous variations of correlated spectral intensities. This suggests the presence of different classes of O–H oscillators characterized by a different out-of-phase dynamical response upon heating. Once again, this appears in agreement with what was hypothesized in our previous 1D FTIR-ATR investigation [42], but is here evidenced without the need of best-fit procedures that imply the use of a variety of parameters to be left free upon iteration.

In particular, the 2DAM of the Coum/SBE-β-CD inclusion complex (Figure 6c) holds two positive vertically squeezed asynchronous regions broadly centered at ~(3430,3268) cm^−1^ and ~(3580,3300) cm^−1^. In addition, the existence of a negative CP at ~(3260,2921) cm^−1^ can be also barely recognized. Nevertheless, since its cross-correlation intensity is too low to be significantly relevant, it will not be discussed.

The shape of CPs at ~(3430,3268) cm^−1^ and ~(3580,3300) cm^−1^ suggests that both regions are actually composed of several individual components, strongly convoluted between each other, associated to the aforementioned different populations of OH oscillators involved in different molecular environments. It is worth noting that the 2DAM of the Coum+SBE-β-CD physical mixture (Figure 6d) shows a slightly different pattern and an overall reduced asynchronous intensity in the entire 2D plane if compared to the corresponding inclusion complex, suggesting a dissimilarity in the nature of the cross-correlations established between the different classes of OH oscillators, as expected.

**Figure 7:**
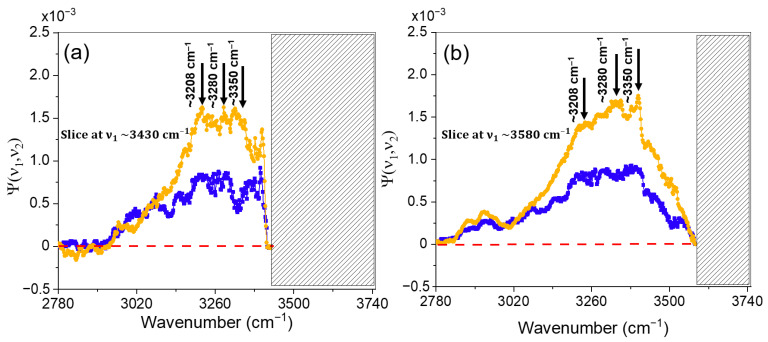
Asynchronous correlation intensity profile calculated along vertical slices characterized by (**a**) $\nu_{1}\;\sim 3430{\;\mathrm{cm}}^{-1}$ and (**b**) $\nu_{1}\;\sim 3580{\;\mathrm{cm}}^{-1}$ for Coum/SBE-β-CD inclusion complex (orange circles) and physical mixture (blue squares). The red dashed line accounts for the $\Psi \left(\nu_{1},\nu_{2}\right)\sim 0$ condition. Thus, positive and negative correlations develop above and below this line. Considering the region of the 2DAM which satisfies the $\nu_{1}>\nu_{2}$ condition (see text for details), information originating from the upper-side portion of the 2D map with respect to the diagonal line must be excluded (black hatched bars). Black arrows indicate $\nu_{2}$ values of interest for our discussion.

In order to better discuss the sequence of events involving all the vibrational modes falling in this spectral region, in Figure 7, we report the comparison of the cross-correlation intensity profiles calculated along the $\nu_{1}\sim \;3430{\;\mathrm{cm}}^{-1}$ and $\nu_{1}\sim \;3580{\;\mathrm{cm}}^{-1}$ vertical slices (yellow dashed-lines in Figure 6c,d) for both the Coum/SBE-β-CD inclusion complex and Coum+SBE-β-CD physical mixture, respectively.

For the interpretation of the different classes of O–H oscillators we recall a well-established assignment [39,42,43,44,45,46]. According to it, contributions at $\nu_{1}\;\sim 3430{\;\mathrm{cm}}^{-1}$ and $\nu_{1}\;\sim 3580{\;\mathrm{cm}}^{-1}$ can be ascribed to the O–H stretching vibrations of primary hydroxyl groups of SBE-β-CD and hydroxyl groups of intracavity water molecules, respectively. As can be observed from Figure 7a,b, both coordinates turned out to be positively correlated with three contributions centered at $\nu_{2}\;\sim 3208{\;\mathrm{cm}}^{-1}$, $\nu_{2}\;\sim 3280{\;\mathrm{cm}}^{-1}$ and $\nu_{2}\;\sim 3350{\;\mathrm{cm}}^{-1}$, which can be attributed to the O–H stretching of secondary hydroxyl groups of SBE-β-CD, hydroxyl groups of Coum and hydroxyl groups of interstitial water molecules, respectively.

According to the Noda’s rules, the following order for the heating process can be reasonable assumed: (i) *T*-induced conformational changes are first experienced by primary OH groups of the cyclodextrin ($\nu_{1}\;\sim 3430{\;\mathrm{cm}}^{-1}$), leading to dynamical variations of the associated O–H stretching mode. (ii) Almost simultaneously, changes in the intracavity water molecules ($\nu_{1}\;\sim 3580{\;\mathrm{cm}}^{-1}$) spectral response can be reasonably hypothesized. (iii) The next step of this chronological evolution involves conformational changes of the secondary OH groups of SBE-β-CD ($\nu_{2}\;\sim 3208{\;\mathrm{cm}}^{-1}$), followed by local re-arrangement of OH groups of Coum ($\nu_{2}\;\sim 3280{\;\mathrm{cm}}^{-1}$) and, lastly, of interstitial water molecules ($\nu_{2}\;\sim 3350{\;\mathrm{cm}}^{-1}$). The interpretation of the proposed chronological sequence affecting the different OH species must take into account the specific electrostatic and steric environment surrounding each class of OH oscillators.

As is well-known, OH primary groups are disposed around the narrower rim of the toroid. Then, on one side, they will be less involved in the establishment of the host-guest interactions driving the complexation process that, as already said, is modeled to occur through the wider rim of the macrocycle [52]. On the other side, if compared to secondary OH groups along the larger side of cyclodextrin, they are allowed to move more freely. Both these factors can justify their fast change rate by temperature enhancement.

At the same time, OH groups of intracavity water molecules were found to experience, upon heating, dynamical changes as a consequence of thermal motion. This occurrence can be justified taking into account that water molecules entrapped within the cyclodextrin molecular cage possess reduced tetrahedral order parameter with respect to those in the bulk material, and hence high enthalpy values. The reduced cooperativity of such water, arising from the presence of four H-bond sites (per molecule) fully available, results in an enhanced orientational/conformational number of degrees of freedom if compared, for example, to water molecules outside the toroidal-shaped structure. This reasonably implies a quick change-rate along an upward temperature trajectory.

The next step involves dynamical alterations of the O–H stretching of secondary OH groups of SBE-β-CD ($\nu_{2}\;\sim 3208{\;\mathrm{cm}}^{-1}$). As a matter of fact, the observed delay in the spectral response can be explained in terms of a statistically significant participation of the secondary OH groups, disposed along the wider rim of the toroid, during the complexation pathway, via host-guest interactions with the C=O groups of Coum, in agreement with what was stated before.

As the final step of the proposed chronological sequence, further increase in temperature was found to induce local/transient reorganization of the hydroxyl species associated to Coum ($\nu_{2}\;\sim 3280{\;\mathrm{cm}}^{-1}$) and interstitial water molecules ($\nu_{2}\;\sim 3350{\;\mathrm{cm}}^{-1}$).

The observed delay is indicative of a strong hindering suffered by these groups, compatible with a previously hypothesized scenario [42,45] according to which complexation induces their rearrangement in long-range, highly-coordinated HB patterns.

Finally, in the case of the Coum+SBE-β-CD physical mixture (see blue squares in Figure 7a,b), the interpretation of the asynchronous correlation intensity profiles, calculated along the two vertical slices ($\nu_{1}\;\sim 3430{\;\mathrm{cm}}^{-1}$ and $\nu_{1}\;\sim 3580{\;\mathrm{cm}}^{-1}$), appears not feasible, due to a non-delineate behavior of the calculated trends and an overall reduced asynchronous cross-correlation intensity between measured spectral responses (with respect to those observed in the case of the Coum/SBE-β-CD inclusion complex). However, the observation of $\Psi \left(\nu_{1},\nu_{2}\right)\neq 0$ values (see blue squares in Figure 7a,b) does not exclude the occurrence of an unsynchronized process followed by different classes of O–H oscillators. This can be reasonably justified by taking into account that both individual components, regardless of whether or not complexation take place, are characterized by a large variety of molecular motions and environments, each of which possess a different susceptibility to external perturbations.

## 3. Materials and Methods

### 3.1. Materials

7,12-dihydorxcoumestane (Coumestrol, Coum, C_15_H_8_O_5_, M.W. = 268.22) was purchased from Sigma-Aldrich Chemie^®^ (Saint-Quentin-Fallavier, France); Captisol^®^ (SBE-β-CD, degree of substitution ≈ 7, M.W. = 2162) was obtained from CyDex, Inc. (Lenexa, KS, USA); all other reagents and solvents used were of analytical grade.

### 3.2. Preparation the Inclusion Complex in Solid Phase

For the preparation of the inclusion complex, the kneading method was used. The required quantities of the active compound and CD were weighed accurately in a ratio of 1:1. The physical mixture (Coum+SBE-β-CD) was mixed for about 10 min, hence, a solvent mixture (water:ethanol, 1:1) was dripped. The mixing continued until complete evaporation for about 1 h. The resulting inclusion complex was dried at room temperature for one day.

### 3.3. FTIR-ATR Measurements

FTIR-ATR spectra were recorded using a Bomem DA8 Fourier transform spectrometer (BOMEM, Saint Laurent, QC, Canada). It was equipped with a Globar source, a KBr beamsplitter and a thermoelectrically cooled deuterated triglycine sulphate (DTGS) detector. Measurements were performed in the 400–4000 cm^−1^ wavenumber range, with a spectral resolution of 4 cm^−1^, and accumulating 100 repetitive scans for each run in order to guarantee a good signal-to-noise ratio as well as high reproducibility. The temperature range explored extended from *T* = 250 K to *T* = 340 K, with a 10-degrees step between each measurement. Samples were positioned in contact with the surface of a single reflectance ATR cell (Golden Gate, equipped with a diamond crystal). The recorded FTIR spectra were properly normalized in order to take into account the effective number of absorbers. No smoothing was applied, whereas baseline adjustment and normalization were performed using the Spectracalc software package GRAMS (Galactic Industries, Salem, NH, USA).

### 3.4. 2-Dimensional Correlation Spectroscopy (2D-COS)

**Scheme 1:**
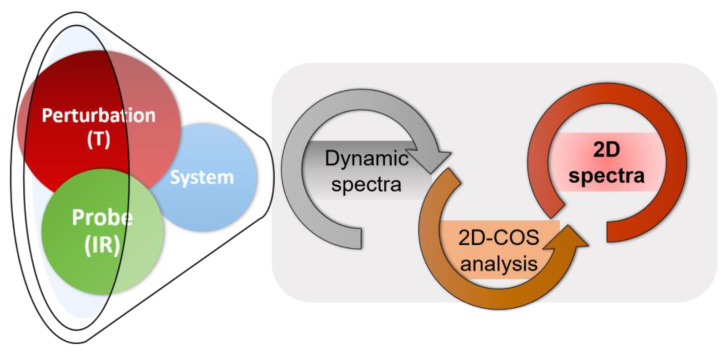
Scheme of the computational strategy adopted for the evaluation of the 2D correlation maps starting from the experimental FTIR-ATR spectra.

The 2D FTIR-ATR correlation spectra were calculated using the OriginLab^®^ software, ver. 2019 (Northampton, MA, USA) following the generalized computational strategy proposed by Noda in 1986 [56,57] (Scheme 1).

Basically, a set of spectra affected by some external perturbation (temperature, in our case) is collected and then transformed in 2-dimensional correlation maps through a well-established cross-correlation analysis. For this purpose, a group of FTIR-ATR dynamic spectra $\tilde{y}(\nu ,T_{i}$), with $T_{i}$ ranging from $T_{min}$ (250 K) and $T_{max}$ (340 K), were first calculated starting from the experimental ones ($y\left(\nu ,T_{i}\right)$) as:

(1) $$\tilde{y}\left(\nu ,T_{i}\right)=\{\begin{array}{l} y\left(\nu ,T_{i}\right)-\bar{y}\left(\nu \right)\;\mathrm{for}\;T_{min}\leq T_{i}\leq T_{max}\\ \;0\;\mathrm{otherwise} \end{array},$$

where $\bar{y}\left(\nu \right)$ denotes the reference spectrum. Commonly, in 2D-COS analysis, there are no strict requirements regarding the choice of the reference spectrum, as it simply suppresses the static contribution coming from the collected FTIR-ATR signals. Usually, the average/stationary or the first/last spectrum of a given spectral series are employed, although any other convenient choice is theoretically acceptable (even $\bar{y}\left(\nu \right)=0$)). For our calculations, the experimental FTIR-ATR spectrum at the lowest temperature (*T* = 250 K) was selected as reference, which yields $\bar{y}\left(\nu \right)=y\left(\nu ,T_{min}\right)=y\left(\nu ,250\;K\right)$. This guaranteed a proper interpretation of the calculated 2D correlation intensities, furnishing information about the simultaneous or unsynchronized sequence of events occurring in the investigated inclusion complex upon upward temperature variations.

According to the numerical approach generally used in 2D-COS analysis [48,49,58], two type of correlation maps can be distinguished, namely synchronous ($\Phi \left(\nu_{1},\nu_{2}\right)$, 2DSMs) and asynchronous ($\Psi \left(\nu_{1},\nu_{2}\right)$, 2DAMs); both providing useful and non-conventional information about the time-dependent order of changes in the molecular behavior along the induced perturbation variable (*T*). Such synchronous and asynchronous 2D correlation maps can be calculated by the following equations [58,59]:

(2) $$\Phi \left(\nu_{1},\nu_{2}\right)=\frac{1}{\varepsilon -1}\overset{\varepsilon }{\underset{i=1}{\sum }}\tilde{y}\left(\nu_{1},T_{i}\right)\times \tilde{y}\left(\nu_{2},T_{i}\right)$$

(3) $$\Psi \left(\nu_{1},\nu_{2}\right)=\frac{1}{\varepsilon -1}\overset{\varepsilon }{\underset{i=1}{\sum }}\tilde{y}\left(\nu_{1},T_{i}\right)\times \overset{\varepsilon }{\underset{j=1}{\sum }}N_{i,j}\times \tilde{y}\left(\nu_{2},T_{j}\right)$$

where *ε* is the dimension of the dynamic spectra dataset collected during the heating process (in our case *ε* = 10) and $N_{i,j}$ denotes an element (*i*-th row and *j*-th column) of the so-called Hilbert–Noda transformation matrix, given as:

(4) $$N_{i,j}=\{\begin{array}{l} 0\;\mathrm{if}\;i=j\\ \frac{1}{\pi \left(j-i\right)}\;\mathrm{if}\;i\neq j \end{array}$$

The 2DSM holds information about the simultaneous or synchronized spectral variations of peak intensities measured at $\nu_{1}$ and $\nu_{2}$. It consists of auto-peaks (APs) developing along the diagonal line ($\nu_{1}=\nu_{2}$, always positive) and cross-peaks (CPs), appearing at off-diagonal positions ($\nu_{1}\neq \nu_{2}$, both positive and negative). The presence of auto-peaks in the 2D plane gives information about the linear evolution of the functional groups conformation, in turn strongly related to the susceptibility of localized species against the applied stimulus (temperature, in our case). However, the interpretation of such peaks is quite straightforward, and does not provide evidence of the existence of correlations, being the AP intensity exclusively related to changes in single FTIR-ATR band intensity. On the contrary, CPs reflect any couple of signals of the experimental FTIR-ATR spectra at $\nu_{1}$ and $\nu_{2}$ which underwent intensity changes in a concordant (increasing or decreasing together) or discordant fashion (otherwise). The 2DAM, on the other side, conveys information about the presence unsynchronized events upon heating, arising from dissimilarity in the rate of change of correlated peak intensities.

In both 2DSM and 2DAM, two orthogonal axes ($\nu_{1}=x$ and $\nu_{2}=y$) of spectral variables (wavenumbers) were adopted to display the 2D plane, while a third axis, normal to the plane, describes the cross-correlation intensity (colors in the 2D spectra). Being both the 2DSMs and 2DAMs specular with respect to the diagonal line having coordinates ($\nu_{1},\nu_{1})$, for the evaluation of the CPs we assumed that $\nu_{1}>\nu_{2}$ (area below the diagonal line).

It is worth noting that, according to the Noda’s rules, the sign of the synchronous/asynchronous CPs furnishes information about the sequence of events followed by the molecular motion along the applied perturbation variable (*T*). Such rules state that if a CP at coordinates $\left(\nu_{1},\nu_{2}\right)$ is positive in both 2DSM and 2DAM ($\Phi \left(\nu_{1},\nu_{2}\right)>0$ and $\Psi \left(\nu_{1},\nu_{2}\right)>0$), then the intensity changes at $\nu_{1}$ faster than at $\nu_{2}$. This means that the corresponding vibrational mode at $\nu_{1}$ “feels” the increase in temperature earlier than that at $\nu_{2}$. An opposite scenario is encountered when $\Phi \left(\nu_{1},\nu_{2}\right)>0$ and $\Psi \left(\nu_{1},\nu_{2}\right)<0$. The aforementioned conditions hold true whenever the 2DSM’s CPs are strictly positive. However, if $\Phi \left(\nu_{1},\nu_{2}\right)<0$, the sequential order of changes must be reversed.

## 4. Conclusions

The complex scenario of events affecting the different functional groups of the inclusion complex based on sulfobutylether-β-cyclodextrin (host) and Coumestrol (guest) (1:1 molar ratio) was here for the first time investigated by a means of a two-dimensional FTIR Correlation Spectroscopy (2D-COS) analysis. In particular, starting from a discrete set of 1D FTIR-ATR spectra, collected during an upward temperature trajectory (between 250 K to 340 K), synchronous and asynchronous 2D correlation maps were properly assessed and discussed. Three different wavenumber ranges of the medium infrared region were focused, namely 960–1320 cm^−1^, 1580–1760 cm^−1^ and 2780–3750 cm^−1^, each of which provided useful and non-conventional information about synchronized and unsynchronized progressions occurring in the original dataset spectral responses upon thermal stimuli.

It is worth noting that the proposed approach opens new possibilities in the view of an in-depth knowledge of the dynamical/conformational features exhibited by materials of interest in the fields of medicine, pharmaceutics and tissue engineering, not easily accessible through conventional spectroscopic methods. Changing the type of analytical probes and external perturbations (such as electromagnetic effects, concentrations, pH and magnetic fields) the role played by the different functional groups of the structure upon different stimuli can be clarified, particularly useful in the definition of an optimized preparation method capable of providing high performance systems for drug delivery applications.

## Data Availability

The data presented in this study are available on request from the corresponding author.

## Sample Availability

Not available.
